# Epigenetic signatures of social status in wild female spotted hyenas (*Crocuta crocuta*)

**DOI:** 10.1038/s42003-024-05926-y

**Published:** 2024-03-28

**Authors:** Colin Vullioud, Sarah Benhaiem, Dorina Meneghini, Moshe Szyf, Yong Shao, Heribert Hofer, Marion L. East, Jörns Fickel, Alexandra Weyrich

**Affiliations:** 1 Department of Evolutionary Genetics, Leibniz-Institute for Zoo and Wildlife Research (IZW), Berlin, Germany; 2 Department of Ecological Dynamics, Leibniz-Institute for Zoo and Wildlife Research (IZW), Berlin, Germany; 3 McGill University, Montreal, Canada; 4 State Key Laboratory of Genetic Resources and Evolution, Kunming Institute of Zoology, Chinese Academy of Sciences, Kunming, China; 5 Leibniz Institute for Zoo and Wildlife Research (IZW), Berlin, Germany; 6 Department of Veterinary Medicine, Freie Universität Berlin, Berlin, Germany; 7 Department of Biology, Chemistry, Pharmacy, Freie Universität Berlin, Berlin, Germany; 8 University of Potsdam, Potsdam, Germany; 9 Present Address: German Centre for Integrative Biodiversity Research (iDiv) Halle-Jena-Leipzig, Leipzig, Germany; 10 Present Address: Universität Leipzig, Leipzig, Germany

**Keywords:** Epigenomics, Molecular ecology, Gene regulation

## Abstract

In mammalian societies, dominance hierarchies translate into inequalities in health, reproductive performance and survival. DNA methylation is thought to mediate the effects of social status on gene expression and phenotypic outcomes, yet a study of social status-specific DNA methylation profiles in different age classes in a wild social mammal is missing. We tested for social status signatures in DNA methylation profiles in wild female spotted hyenas (*Crocuta crocuta*), cubs and adults, using non-invasively collected gut epithelium samples. In spotted hyena clans, female social status influences access to resources, foraging behavior, health, reproductive performance and survival. We identified 149 differentially methylated regions between 42 high- and low-ranking female spotted hyenas (cubs and adults). Differentially methylated genes were associated with energy conversion, immune function, glutamate receptor signalling and ion transport. Our results provide evidence that socio-environmental inequalities are reflected at the molecular level in cubs and adults in a wild social mammal.

## Abstract (web summary)

An epigenetic study on free-ranging spotted hyenas (*Crocuta crocuta*) suggests that genes are differentially methylated between high-ranking and low-ranking females (cubs and adults) which may reflect rank-specific differences in access to resources.

## Introduction

Dominance hierarchies have evolved in numerous group-living mammal species and are generally characterised by preferential access to limited resources for dominant (socially high-ranking) individuals over subordinate (low-ranking) ones^1^. Submission by subordinate individuals to more dominant group members is a convention that reduces the need for costly aggression during encounters. In many mammal societies, the quality of life at the top of a hierarchy considerably differs from that at the bottom (e.g., ref. ^2,3^). High social status provides priority of access to critical resources such as food or mates, a privilege that affects an individual’s life history^4^, by ultimately increasing its survival and lifetime reproductive success, compared to individuals of low social status^5^. Importantly, these social status-related differences in life-history traits are accompanied by differences in physiological processes and health^2,6^. Given the significant effects of social status on life-history trajectories, survival, and health of individuals in social mammals, including humans (e.g., ref. ^7^), our study aims to investigate whether there are distinct DNA methylation signatures associated with social inequalities experienced by high- and low-ranking individuals in a socially structured wild mammal society.

Social status is a trait that emerges from an individual’s interactions with their social environment^8,9^. In nepotistic societies, where individuals typically benefit from supporting their relatives, offspring receive social support from their mothers during interactions with group members and learn thereby that they can dominate all individuals who are submissive to their mother, but are subordinate to those that their mother is submissive to (e.g., in cercopithecine primates such as yellow baboons *Papio cynocephalus*^3^ or spotted hyenas *Crocuta* *crocuta*^10,11^). In species with female philopatry, this maternal rank ‘inheritance’ results in the youngest daughter holding a rank immediately below that held by her mother and above that of her older sisters^12^. By this mechanism, social status is passed to the next generation, and high-born offspring gain privileges associated with their mother’s high rank (a ‘silver spoon’ effect)^13^. Therefore, in these societies, social status is both stable and relatively predictable because it is determined by family relationships and behavioral conventions (e.g., ref. ^3,14^), and the effects of social status on life-history trajectories and health are typically passed across generations (e.g., in spotted hyenas^10,11,15–17^).

We aim to investigate whether molecular signatures of social status can be identified in a population of individually known female spotted hyenas (hereafter ‘hyenas’), focusing on both adults and juveniles during their first year of life (termed cubs). Hyena clans are fission-fusion societies in which adult females and their offspring socially dominate immigrant breeding males, and there is a strict linear dominance hierarchy among adult females^18^. In our study population in the Serengeti National Park, in northern Tanzania, hyenas older than approximately one year regularly ‘commute’ long distances, i.e., leave their clan territory and travel up to 70 km to forage in areas containing large aggregations of migratory herbivores, their main prey, before returning to their clan territories^19^. As social status determines the priority of access to resources within clan territories, high-ranking females less often leave on long-distance commuting trips than low-ranking ones^15,19,20^. As a consequence, high-ranking females are more often present in their clan territories, and, therefore, more frequently nurse their offspring at the clan communal den within the territory^15,20^. Their milk-dependent cubs thus disproportionately benefit at this early stage in their life; by growing faster and having a higher chance to survive to adulthood. Daughters of high-ranking females also start reproducing at an earlier age^15,16^. The positive effects of high social status on performance and fitness in both juveniles and adults have also been reported in other study populations (e.g., ref. ^17,21,22^).

As in other social mammals, social status in female hyenas influences health and resistance against pathogen infection. Because immune responses are very resource-demanding, the lower access to resources by low-ranking females is thought to compromise their immune responses to infections^23^. For instance, low-ranking females have higher infection loads of the gastrointestinal hookworm *Ancylostoma*^24^ and lower levels of two circulating immune defence factors, complement-mediated bacterial killing capacity (BKC) and total IgM^23^. In addition, the offspring of low-ranking females were found to have a lower probability of surviving infections than the offspring of high-ranking females, as they had high loads of *Ancylostoma*^11^ or systemic infection with a highly virulent strain of canine distemper virus^10^. Social status in a range of species may also influence glucocorticoid (GC) concentrations, which can be higher in subordinates than in dominants when social hierarchies are stable^2,25^. In Serengeti hyenas, a relationship was found between social status and fecal GC concentrations among non-lactating female hyenas during periods of social stability (i.e., in the absence of coups), with higher levels of GC in low- than high-ranking females^26^.

Physiological processes are mediated by a number of processes, including gene regulation. Gene regulation in turn is partly regulated by epigenetic mechanisms such as DNA methylation, histone modification, and small non-coding RNAs^27^. These molecular mechanisms contribute to the translation of environmental conditions into changes in gene activity, without modifying the DNA sequence^28,29^. The early developmental period is a phase of high plasticity and dynamic gene expression (e.g., during cell differentiation,^30^). In this phase, most epigenetic patterns are set during cell differentiation as characteristics of cell types. Methylation patterns are maintained after DNA replication, during cell mitosis and germ cell meiosis^31^ and remain stable throughout an individual’s lifespan and even partially across generations^29,32,33^. Even so, substantial amounts of DNA are de novo methylated and demethylated and respond to changes in environmental conditions, e.g., diet (wild guinea pigs *Cavia aperea*^34^, yellow baboons^35^), infection, and immune regulation (rhesus macaques *Macaca mulatta*^36^), physical exercise (humans)^37^ or social stress (rats^,38,39^, rhesus macaques^40^).

Social environments are known to influence DNA methylation^6,36,41^. For instance, in yellow baboons DNA methylation changes were detected in immune-related genes in blood from adult males that fight for social status, but not among females whose social status is based on kin-directed nepotism^42^. In captive rhesus macaques, DNA methylation patterns differed significantly between high-ranking and low-ranking females in two different tissues: (1) in the placenta, in genes involved in cellular growth and proliferation, apoptosis, and transport of molecules^40^, and (2) in peripheral blood cells, where social rank also influenced the expression of genes important for immune surveillance and defence, glucocorticoid receptor (GR)-mediated signalling and proinflammatory responses^6^.

In free-ranging hyenas in the Maasai Mara National Reserve, Kenya, global DNA methylation (the percentage of methylated DNA in total DNA; global %CCGG) in whole blood samples from cubs (<1 year) was influenced by maternal rank, with a 2.75% higher CCGG methylation percentage in high-ranking cubs^43^. In contrast, maternal rank during the year of birth of the offspring did not influence global CCGG methylation in subadults (1–2 years of age) or adults (>2 years), even after controlling for a potential effect of an individual’s own social status. This suggests that at the global CCGG methylation level, the effect of social status in early life may not persist^43^. In a subsequent study of the same population, an epigenome-wide approach based on whole blood samples of 30 juveniles (11–27 months) showed an association between the time spent in close physical proximity between mothers and their offspring, i.e., a measure of maternal care, and DNA methylation profiles but no direct effect of maternal rank on methylation patterns^44^. Together, these results on hyenas suggest that, if anything, social status influences the epigenome only among cubs, i.e., during the early life period.

Importantly, many of the studies investigating the effects of social status on the epigenome were based on samples collected from captive animals (e.g., ref. ^6,36,40^). Even if dominance hierarchies exist in captivity, the experiences of high- or low-rank individuals are likely to differ considerably from those in free-ranging populations. In addition, all studies on this topic used samples containing DNA extracted from blood, which were collected invasively. Even though blood, brain, and liver samples are often used in epigenetic studies, the intestinal epithelial cell layer, which is an important defence tissue of the mammalian immune system^45,46^, can be a relevant tissue to study the effects of social interactions on the epigenome (e.g., ref. ^32,40,47^). Invasive or lethal approaches are required to obtain blood, brain, and/or liver tissues. This poses ethical considerations and might cause disturbances that influence results and limit or even prevent the applicability of epigenetic studies in wildlife populations (e.g., ref. ^48^), particularly in protected areas were interventions are strictly limited or prohibited. Hence, there is a need to develop and validate approaches based on non-invasive samples. To our knowledge, no previous study has attempted to investigate the effects of social status on DNA methylation in free-ranging wildlife populations using samples collected non-invasively. Finally, we assessed both the robustness and discriminative power of status-specific methylation differences to determine if social status influences DNA methylation across the genome.

Here, we report an in-depth assessment of the relationship between social status and DNA methylation in individually known free-ranging hyenas, using non-invasively collected samples from both female cubs and adults. We hypothesised that DNA methylation mediates the effects of social status on health, behavior (incl. commuting and foraging), and fitness from an early age in female hyenas, and predicted that social status (here between two main social classes ‘high-ranking’ *vs*. ‘low-ranking’) is associated with status-specific DNA methylation signatures in both female cubs and adults. More specifically, we expected differential DNA methylation in genes involved in metabolism, energy conversion, and immune function. We further assessed whether status-specific DNA methylation signatures discriminated between high-ranking and low-ranking females.

## Results

**Fig. 1:**
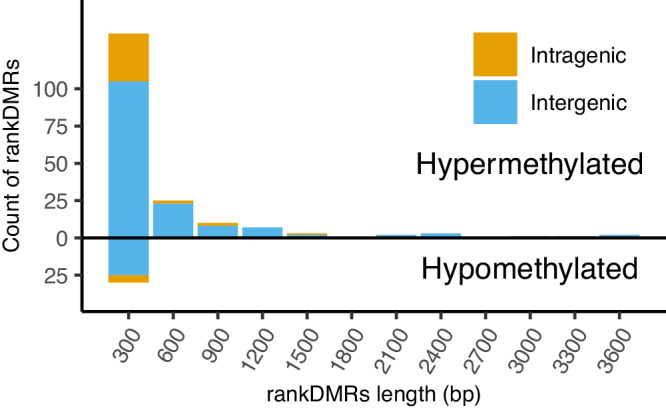
Hypermethylated and hypomethylated regions in low-ranking female hyena cubs and adults according to their length. Number of rankDMRs, hypermethylated (above 0) and hypomethylated (below 0) in low-ranking female cubs (*n* = 24) and adults (*n* = 18) sorted by their length (in base pair (bp)). Intergenic regions are in blue, and intragenic regions are in orange. RankDMRs were at least 300 bp long (size of one window and mean fragment size for sequencing). Adjacent rankDMRs with change in the same direction (either hypermethylated or hypomethylated) were merged, resulting in longer DMRs.

**Fig. 2:**
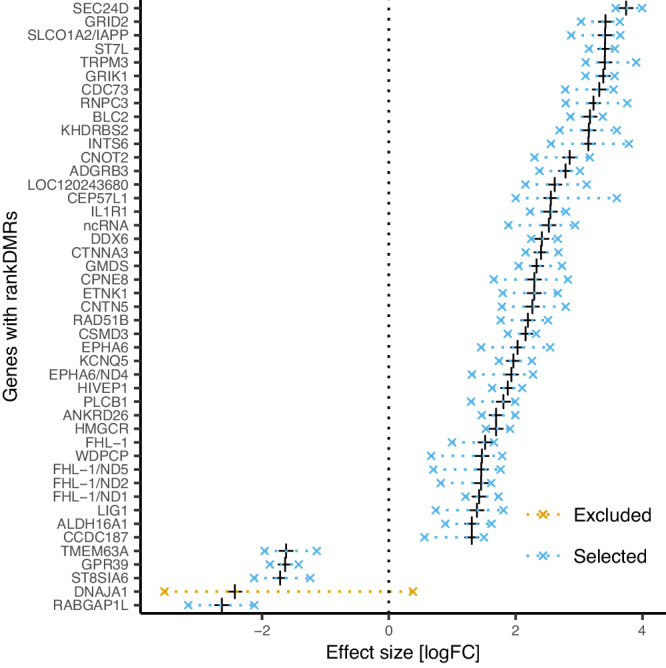
DNA methylation differences between high- and low-ranking female hyenas in annotated genes according to their fold-change, hypermethylation, and hypomethylation and effect range. The black cross is the observed log_2_ fold-change (logFC) value in the full dataset (*n*_rankDMRs_ = 179, *n*_windows_ = 321). The *x* symbols and the dashed lines mark the logFC range across the subsets. Blue dashed lines indicate that the direction of the effect (hypermethylation vs. hypomethylation) across the subsets was coherent (selected rankDMRs), and the orange dashed line indicates that the effect was incoherent (excluded rankDMRs). The rankDMR overlapping the gene encoding for *DNAJA1* was not used for the downstream analysis. See Supplementary Fig. 3 for equivalent results in intergenic regions.

To assess if social status influences DNA methylation patterns we extracted DNA from gut epithelium cells removed from fecal samples that were collected soon after deposition from individually known females (Supplementary Fig. 1), including 18 high-ranking females (9 cubs, 9 adults) and 24 low-ranking females (15 cubs, 9 adults) (Supplementary Results, Supplementary Fig. 2a). Cubs were younger than one year and adults were older than two years (Supplementary Fig. 2b, Supplementary Table 2). We then sequenced the methylated reads after methylation capture with methyl-binding domain protein 2 (MBD-Seq). We used a negative binomial regression to model the read counts of 300 bp genomic sliding windows (mean fragment size sequenced) as a function of female social status category (high-ranking *vs*. low-ranking), while controlling for age class (cub *vs*. adult). Using this methylation analysis, we identified 179 differentially methylated regions (DMRs) between high-ranking and low-ranking females (hereafter ‘rankDMRs’). RankDMRs consist of one or more adjacent windows for which the effect (log_2_ fold-change (logFC)) of social status on the number of methylated reads was statistically significant after correction for multiple testing (false discovery rate (FDR) ≤ 0.05, Fig. 1). Lengths of rankDMRs ranged from 300 bp (1 window) to 3600 bp (12 adjacent windows) (Fig. 2).

To map rankDMRs to functional regions, we used gene annotations from NCBI and extended them by adding TSS annotations and defining promoters as the 2 kb upstream of the TSS for the recently published spotted hyena genome^49^. Forty-two rankDMRs overlapped with at least one intragenic region (incl. promoter, transcription start site (TSS), start-codon, exon, intron, stop-codon), and 137 were located in intergenic regions (Table 1).

**Table 1:** RankDMRs overlapping with annotated regions

Promoter	Transcription start site	Start-codon	Exon	Intron	Stop-codon	Intergenic region
2	1	1	6	39	2	137

Most rankDMRs were hypermethylated in low-ranking individuals and overlapped intergenic regions (Fig. 1). The longest rankDMRs overlapping genes had a length of 1500 bp, including the genes (*ND1*, *ND5*, *FHL-1*; Supplementary Table 3).

The FDR procedure corrects for multiple testing but does not address potential overfitting of the model. We assessed whether these rankDMRs were coherently differentially methylated by subsampling the 42 samples (Supplementary Table 1) into 6 subsets with each containing 35 individuals. To do so, we randomly split the individuals into six groups and removed one group at a time. We then analyzed each subset using the same methylation analysis as for the full dataset and kept the *p* values and effect sizes (log_2_ fold-change, logFC) of the positions of the rankDMRs detected in the full dataset. For further analysis, we filtered for rankDMRs for which all windows across all subsets had a *p* value ≤ 0.05 and a coherent effect direction (e.g., all subsets showed an hypermethylation or hypomethylation), hereafter termed ‘selected rankDMRs’. Of the 179 rankDMRs, 30 did not fit our criterion for inclusion in further analyses, revealing a strong dependency on a few samples. These 30 rankDMRs were removed from downstream analysis (termed ‘excluded rankDMRs’). Of the 42 rankDMRs overlapping 45 genes only one (overlapping the gene *DNAJA1*) was removed (Fig. 2).

**Fig. 3:**
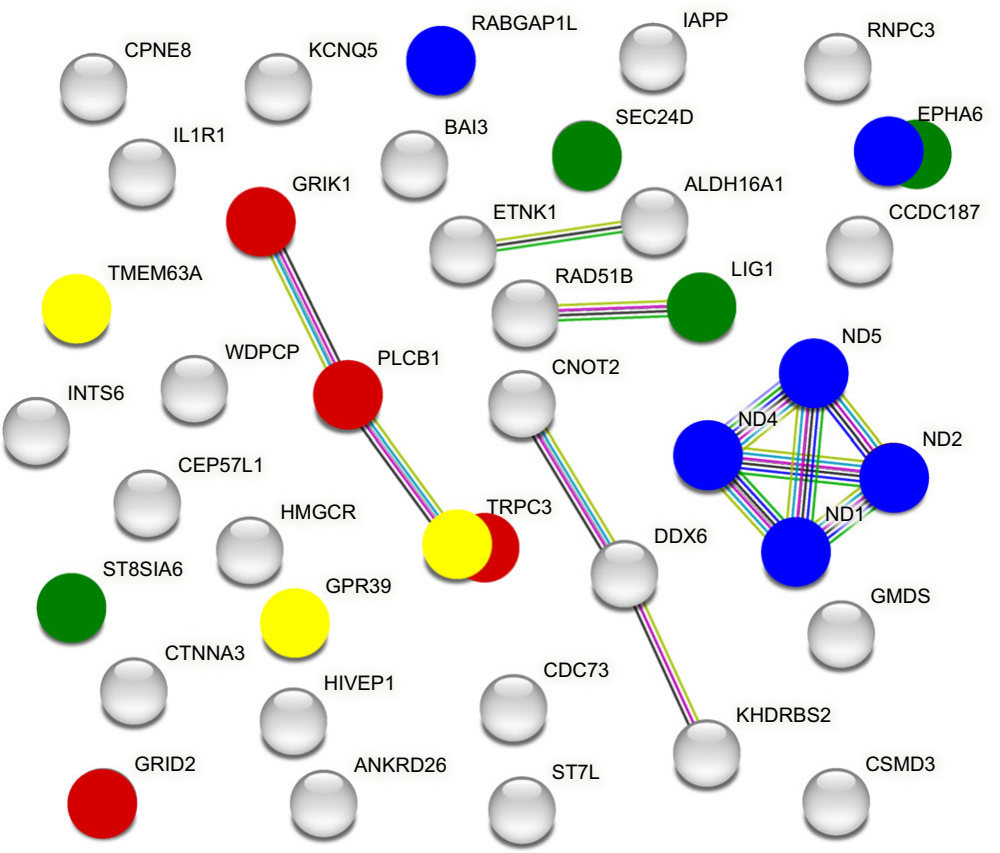
STRING-network of genes differentially methylated between high- and low-ranking female hyenas. 44 differentially methylated genes were involved in: energy conversion (blue dots), glutamate receptor signalling (red dots), immune responses (green dots) and ion transportation (yellow dots), or other functions (grey dots). Overlapping dots with different colours represent proteins that belong to more than one pathway. The network is based on the set of 44 coherent genes, and resulted in 39 gene-encoding proteins (dots) (using *Felis catus* as reference) that had significantly more interactions (12 edges) than expected (4 edges) for a *random* set of proteins of the same size and degree distribution drawn from the genome (*p* value = 0.002). The network indicates that the proteins are at least partially functionally connected.

We performed a gene network analysis on the ‘selected rankDMR’ (*n* = 41) that overlapped with 44 genes (40 nuclear, 4 mitochondrial). Gene network analyses identify genes with similar biological functions (according to KEGG and GOterm databases). The analysis resulted in four main pathways with functions in energy conversion, glutamate receptor signalling, immune response, and ion transport (Fig. 3).

The gene with the biggest fold-change is SEC24 Homologue D COPII Coat Complex Component (*SEC24D*) which is an immune-relevant gene, as well as DNA Ligase 1 (LIG1), ST8 Alpha-N-Acetyl-Neuraminide Alpha-2,8-Sialyltransferase 6 (*ST8SIA6*), and *EPHA6* (Fig. 3, green dots). Several genes involved in energy conversion included nuclear genes such as Ephrin type-A receptor 6 (*EPHA6*) and Rab GTPase-activating protein 1-like (*RAPGAP1L*), as well as mitochondrial genes such as NADH dehydrogenase subunits *ND1*, *ND2*, *ND4* and *ND5* (Fig. 3, blue dots), all of which were hypermethylated in low-ranking females. ND proteins are essential for ATP production and oxidative phosphorylation in the mitochondrial respiratory chain. We detected rankDMRs in ion transport genes, e.g., G-protein-coupled receptor 39 (*GPR39*), transient receptor potential cation channel subfamily C member 3 (*TRPC3*), and transmembrane protein 63 A (*TMEM63A*) (Fig. 3, yellow dots). The glutamate receptor signalling pathway (Fig. 3, red dots) is represented by the genes Glutamate ionotropic receptor kainate 1 (*GRIK1*), Glutamate ionotropic receptor delta type subunit 2 (*GRID2*), Phospholipase C, beta 1 (phosphoinositide-specific) (*PLCB1*), and Transient receptor potential cation channel subfamily M member 3 (*TRPC3*) (Fig. 3, red dots). The glutamate receptor signalling pathway is important in the gut-brain axis, enabling exchange of information between the gut and brain and providing systemic energy.

To address the possibility that one age class drives the results, we looked at the effect of rank on DNA methylation within the 179 rankDMRs in cubs and adults separately (Supplementary Fig. 4). In these genomic regions, the effect was positively correlated (Pearson’s product-moment cor= 0.47; Supplementary Fig. 4a), and this was significantly higher (*p* < 0.001; Supplementary Fig. 4b) than in other genomic regions. Seven genes (*WDPCP*, *EPHA6*, *ND1*, *ND2*, *ND4*, *ND5*, *FHL-1*; Supplementary Fig. 4c) showed a negative effect (hypomethylation in low-ranking) in cubs but a positive one (hypermethylation in low-ranking) in adults. As the effects in these genes were all positive (hypermethylated) in the main model (see Fig. 3), this indicates that in those regions, the combined effect was mostly driven by the adult group. We then assessed how accurately the observed rankDMRs could discriminate between high- and low-ranking female hyenas. In other words, whether the social status of a female could be predicted by its methylation pattern at specific genomic regions. To do so, we trained four random forest (RFs) classifiers on different subsets of the observed DMRs, including total, selected, and excluded rankDMRs as well as mean DNA methylation (estimated as the sum of read counts for all windows (library size after normalisation) per individual).

Selected (*n* = 149), and total (*n* = 179) rankDMRs showed a similar discriminative power of ~80% (Table 2). In contrast, excluded rankDMRs (*n* = 30) performed poorly as did ‘mean DNA methylation’ with a discriminative power at about chance level. Looking at curated rankDMRs thus improved the discriminative power of the random forest classifier well above the chance level.

**Table 2:** Discriminative power of DMRs between high- and low-ranking female hyenass

Variables used for prediction ^*^	oob error range [%]
Total rankDMRs	14.3–23.8
Selected rankDMRs	14.3–23.8
Excluded rankDMRs	42.9–57.1
Mean DNA methylation	45.1–52.4

*A random forest classifier is a learning procedure that operates by creating decision trees using a subset of the variables and a subset of the samples. The samples not used to build a tree (out-of bag-samples) are used as a test-set for the classification outcome of this tree. The average classification error on the out-of-bag-samples (oob-error) provides an estimate of the general error rate. We use oob-error as a proxy for the discriminative power of a group of rankDMRs. While oob-errors are insightful in this comparative setting, their absolute value should be taken with caution because; 1. we used the same dataset to select the rankDMR and to train the random forest classifier, 2. we did not fine-tune the hyperparameters of the model, and 3. did not investigate other learning approaches.

The table shows the range of mean classification errors on out-of-bag-samples (oob) in 1000 repetitions for different sets of variables.

**Fig. 4:**
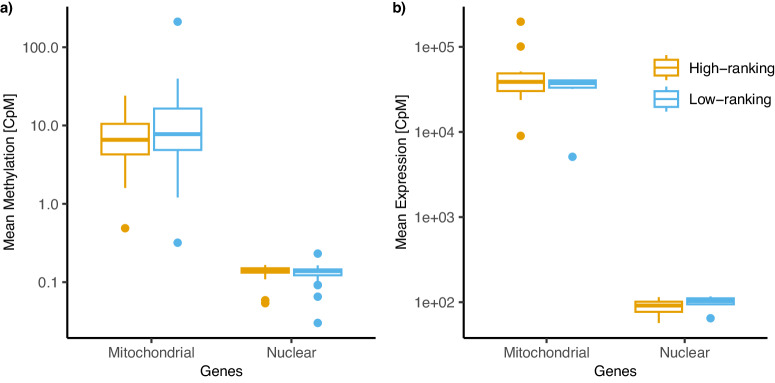
Gene activity in high- and low-ranking female hyenas of mitochondrial vs. nuclear DNA and RNA reads. **a** Mean DNA methylation of mitochondrial and nuclear DNA per window and individual in count per million bases (CpM) and **b** mean RNA expression per gene and individual in CpM, indicate greater abundance of methylation and expression of mitochondrial than nuclear DNA and RNA in adult females (*n*_high ranking_ = 10 and *n*_low ranking_ = 6). The upper and lower hinges correspond to the first and third quartiles, and the horizontal lines corresponds to the median. The upper whisker extends from the hinge to the highest value that is within 1.5 × inter-quartile range (IQR) of the hinge, or the distance between the first and third quartiles. The lower whisker extends from the hinge to the lowest value within 1.5 × IQR of the hinge. Data beyond the end of the whiskers are plotted as dots.

Finally, to test for the possibility that the rankDMRs from mitochondrial genes are nuclear mitochondrial DNA copies, which are inactive mitochondrial gene copies integrated into the nuclear genome^50^, we investigated gene activity using a transcriptome analysis (RNA-Seq) in 16 gut epithelium samples from different adult females (10 high-ranking, 6 low-ranking). We detected significantly higher methylation levels of mitochondrial genes (Wilcoxon signed rank test, Z = 2.58, *p* = 0.009) than of nuclear genes, and higher expression of these mitochondrial genes (*Z* = 4.94, *p* < 0.001) than in nuclear genes (Fig. 4). The expression of the mt-genes supported the functional activity and relevance of these mitochondrial genes. The difference in mean DNA methylation and gene expression was not significantly different between high-ranking and low-ranking adult females (DNA; *Z* = 0.099, *p* = 0.920 and RNA; *Z* = −0.156, *p* = 0.893). Differences between mitochondrial and nuclear genes can originate from both differential methylation and expression levels, and/or differences in the ratio of mitochondrial to nuclear genes.

## Discussion

In many social mammals, the quality of life at the top of a dominance hierarchy considerably differs from that at the bottom (e.g., ref. ^2,3^). In hyenas, the social status of females in their clan’s linear dominance hierarchy is a key determinant of their degree of access to resources, social behavior, commuting, health, and reproductive performance, and these rank-related differences emerge early in life (e.g., ^16,17^). Our results from non-invasively collected gut epithelium cells identified 149 differentially methylated regions (rankDMRs) between high- and low-ranking female hyenas in the Serengeti National Park. More DMRs were hypermethylated in low- than in high-ranking females, after controlling for the effect of age class (cub and adult). RankDMRs in 44 genes were involved in pathways related to energy conversion, immune response, glutamate receptor signalling, and ion transportation. The rankDMRs were located in nuclear DNA (ncDNA) and mitochondrial DNA (mtDNA). The two mitochondrial genes *ND1* and *ND2* were methylated in their promoter regions. While the majority and the longest rankDMRs (up to 3600 bp) were observed in intergenic locations, we detected three genes with overlapping rankDMRs of 1500 bp (5 adjacent 300 bp windows), including *ND1*, *ND5*, together with the Complement factor H-like (*FHL-1*) gene, encoding for a protein with an essential role in defence to microbial infections. Overall, our results indicate that social status is reflected in specific DNA methylation signatures in both cub and adult female hyenas in genes associated with immune processes, energy conversion, glutamate receptor signalling and and ion transport. Behavioral processes that determine social status and life-history trajectories also affect epigenetic signatures in female hyenas.

A previous study on wild hyenas found an effect of maternal social status on global DNA methylation percentage (global %CCGG) in cubs (<1 year), but not in subadults (1–2 years) and adults (>24 months)^43^. This study also revealed global methylation ~2.75% higher in cubs of high-ranking mothers than in those of low-ranking ones. A more recent study using an epigenome-wide approach revealed no effect of maternal rank on differentially methylated CpGs of juveniles aged between 11 and 27 months^44^. Instead, measures of maternal care and social connections early in life influenced global DNA methylation assessed in adulthood^44^. In contrast to these two studies, we found an effect of social status in both cub and adult female hyenas; i.e., 149 selected DMRs that differed between high- and low-ranking females, and a higher methylation in low-ranking females. These different findings in the same species can be explained to some extent by different resolution of methodological approaches; i.e., global %CCGG results in a single value for the whole genome methylation, whereas mRRBS^44^ and MDB-Seq (used here) provide a higher resolution of specific regions, and enabled identification of genome-wide DNA methylation signatures. The different findings between Laubach et al.^44^ and our study may also be explained by the use of different sample materials (whole blood *vs*. gut epithelium cells) and age classes (juveniles *vs*. cubs and adults). More importantly, our study benefited from the novel spotted hyena genomic reference sequence^49^, which we used to map our MBD-Seq data, in contrast to the human and cat (*Felis catus*) reference genomes used previously^44^.

Cell differentiation processes during early development lead to cell-type-specific methylation patterns^30,51^. As mentioned by Laubach et al.^44^, cell-type heterogeneity in whole blood might be a source of variation in DNA methylation. Thus, a single cell type (and/or a stable combination of cell types) is optimal to study DNA methylation. Here we used epithelial cells taken from the feces surface, consisting of mainly one cell type. These cells coat the surface of matter or adhere to mucus, which coats some feces on its pass through the hindgut. The intestinal epithelium cells, which play an important immunological role, are a physical and biochemical barrier in preventing pathogens and other harmful substances entering the body^45,52^. In wildlife epigenetic studies, a non-invasively collected sample material consisting of mainly one cell type is of great advantage and can be applied to a range of mammals.

Among the genes differentially methylated between high- and low-ranking females, we found a relatively large number of genes involved in energy conversion (*ND1*, *ND2*, *ND4*, *ND5*, *FHL-1,* and *EPHA6*, Fig. 3). We suspect that this result is mostly driven by the greater use of long-distance foraging trips by low- than high-ranking females, which monopolise resources in their clan territory. A recent analysis based on >30 years of data in Serengeti hyenas showed that low-ranking females with dependent cubs younger than six months spend significantly less time at clan communal dens in their clan territories^20^ and thus more time commuting long distances of up to 70 km to foraging areas containing high densities of migratory prey^19^. This result is consistent with previous research showing that low-ranking females have longer mean absence intervals from their territories and are generally less present in their territories^15,19^. Interestingly, these genes were hypermethylated in low-ranking in comparison to high-ranking females in adults, but hypomethylated in cubs. Those two findings could indicate an adjustment of low-ranking adult females to great energetic costs of frequent long-distance commuting, a behavior not displayed by cubs.

The physiological consequences of such hypermethylation in low-ranking females remain to be investigated. The finding of rankDMRs located in mtDNA is novel, because until recently mtDNA had been thought to be unmethylated^53,54^. Recent findings, however have revealed that mtDNA can be methylated and have specific cytosine methylation patterns^55–58^. Here, we detected promoter methylation in two mt-genes ND1 and ND2, which is a main genomic region of DNA methylation to regulate gene expression in mammalian genomes^59^. The function of promoter methylation in mitochondria remains unclear^60^. As the number of mitochondria is high in gut epithelium cells, our findings can result either from (1) higher mtDNA methylation in low-ranking than high-ranking females, or (2) a higher abundance of mitochondria in low-ranking than high-ranking individuals. Both reasons suggest again a potential difference in energy conversion between high- and low-ranking female hyenas. It would be interesting to compare these rank differences with other free-ranging populations where prey abundance in clan territories is generally higher and commuting distances shorter^21,61^.

Interestingly, the gene with the highest range of effect size of rankDMRs (SEC24 Homologue D, COPII Coat Complex Component (*SEC24D*)), and the gene with the longest rankDMR (Complement factor H-like (*FHL-1*)) were relevant to immunity (Figs. 2, 3, Supplementary Table 3). We expected differences in genes relevant to immunity, as previous research from our group and that of others suggested high-ranking hyenas are less prone to both gastrointestinal parasites and pathogens of infectious diseases at different ages^10,11,23,24^. Because maintaining a competent immune system is very resource-demanding, the lower access to resources by low-ranking females is thought to compromise their immune responses to infections^23^. It is unclear whether DNA methylation differences in such genes may, to some extent, help low-ranking females to cope with nutrition restriction and pathogen pressures, for instance, by utilising energy stores more efficiently. Several epigenetic studies on primates in captivity similarly revealed methylation and gene expression differences in animals of different ranks in genes involved in immune function (yellow baboons^42^, rhesus macaques^36^).

DNA methylation patterns at the putative glucocorticoid receptor (*GR*) in peripheral blood mononuclear cells have been reported to differ in the context of social classes in captive adult female rhesus macaques^6^, in relation to the level of grooming behavior of rat mothers^38^ and in humans abused during childhood^47^. Even so, the relationship between maternal care and changes in GR methylation patterns has not been replicated in a study using rodent hippocampus cells^62^. Similarly, our result from gut epithelium cells revealed no evidence of DNA methylation differences in stress-related genes between high- and low-ranking female adults and cubs. Intestinal epithelial cells respond to physiological stressors^46,63^ and are a tissue suitable for investigating the effects of social environments on DNA methylation patterns. While an increasing number of studies on free-ranging social mammals show that responses to social environments are reflected in the epigenome, to our knowledge none had previously demonstrated an effect of social status on DNA methylation profiles in both cubs and adults in a free-ranging population. In addition, all previous studies were based on samples collected invasively (i.e., blood samples) either in captivity under experimental conditions^6,40^, or in the wild after capture of animals^42–44^. As capture and immobilisation procedures may alter individual behavior and physiology (i.e., glucocorticoid (GC) levels in particular), these anthropogenic effects could potentially affect the epigenome.

Our study presents for the first time the feasibility, and as such proof of principle, of performing a high throughput DNA methylation analysis using gut epithelium cells collected non-invasively from fecal samples from a wild mammal. Non-invasive sampling provides several advantages over invasive sampling. Firstly, it avoids the physiological stress of capture process that could alter GC measures and DNA methylation profiles. Secondly, in most mammals the collection of fresh fecal samples is easier (more accessible) and cheaper than that of invasive samples. Therefore, more samples can be obtained at lower costs and without specific capture and medical instruments, or veterinary training. This provides the opportunity for more longitudinal study designs. Thirdly, it allows the collection of samples without direct contact with the animal and thus allows to reach animals that are inaccessible via invasive sampling. For example, the youngest sampled animal in our study was two months old (Supplementary Table 1), an age at which capture by any method would cause substantial disturbance at clan communal dens. For the same reason, it can provide field biologists with additional methodological tools and broaden the scope of epigenetic analysis to fields such as behavioral ecology, for instance by monitoring the age distribution of populations with epigenetic clock^64^. Finally, epigenetic analysis of fecal material can be combined with other information present in the feces such as genetic^22,65^, endocrine^66^ and immune measures, or parasite load and infection status^11,67^.

These advantages come at the cost of quality. DNA quality of materials (e.g., feces, hair, epithelium cells) is usually lower than in tissue samples collected invasively (e.g., blood, organs). Some cells collected from feces may not represent living gut cells, and can as such, be less informative about biological processes. Even so, they may be of sufficient quality to investigate some genetic questions^68,69^. Gut epithelium collected from feces can contain beside hyena DNA, DNA from gut bacteria, fungi, parasites, and of prey species recently consumed^70^. The MBD2 capture approach addressed this issue, because MBD2 specifically binds to mCpG, a position that is mostly methylated in mammals, but not in bacteria^71^. By this, we enriched for methylated and mammalian DNA simultaneously, and sequenced mainly hyena DNA. The presence of long rankDMRs of up to 3600 bp served as proxy for high DNA quality and reliable MBD-captured DNA sequencing, because gaps in sequencing would prevent the generation of such long stretches of DNA.

Fresh liver and brain are tissues generally used in laboratory studies on the epigenetic effects of physiological stress and behavior. As these tissues are obtained post mortem they cannot be obtained from large mammals within protected areas established to preserve wildlife and can thus only be sampled from animals kept in captivity^72^. In this condition, social dynamics are, however, not necessarily reflecting the natural processes occurring in wild populations.

Our study, like others in the field of ecological epigenetics on wildlife populations, suffers from a relatively small sample size. Before making inferential interpretations (pathway analysis), we aimed to extract the most information from our data and filtered the DMRs to improve the quality of the epigenetic data used in our analysis (selected rankDMRs). To test which of the rankDMRs would be trustworthy, we performed a resampling approach that identified 30 rankDMRs (of 179) that were not coherently hypermethylated or hypomethylated, or showed a large variation in effect size (Fig. 2, Supplementary Fig. 3), which we excluded for our downstream analysis (excluded rankDMRs). The use of random forest classifiers to compare different findings on an objective metric (discriminative power) allows uncovering the potential danger of relying on a single variable or a single method to draw inference, such as the mean methylation level (indicated by an oob of 50%). In other terms, we believe that these methods can minimise misleading inferences. The almost infinite ways to question and analyze the relationship between socio-environmental phenomena and molecular mechanisms (“forking path” problem^73^) raises concern about the reproducibility of our and other findings. This underlines the importance of establishing an inventory of basic epigenetic patterns, across species and tissues and a more fundamental descriptive representation of interesting genomic regions of epigenetic stability and flexibility^74^.

The 149 robust rankDMRs detected here suggest that social status gets “under the skin” of individual hyenas, as suggested for baboons and macaques^6,36,42^. Whether the observed 149 methylation events accurately reflect social status, and can be generalized, remains unknown, yet they represent a first set of status biomarkers for wildlife to be tested in other hyena populations and potentially other social species^72^. These biomarkers of social status in hyenas could aid in determining the social status class of unknown individuals, which are not monitored as part of long-term individual-based projects, such as in roadkill studies. In addition, our study provides a database of the genome-wide methylation data of 42 hyenas, including a custom-made annotation (NCBI) for the newly available hyena genome^49^. It is noteworthy, that besides other factors, genetic variation and relatedness can affect epigenetic patterns, and were not considered in our study design. Investigation of a broader range of factors requires data from a larger number of individuals, but the current high costs of analysis precluded this, thereby limiting the scope of our statistical analysis. As such, we hope wildlife scientists and conservation managers may benefit from the current study, which illustrates that epigenetics can be studied in wild mammals using non-invasive tissue collection, and a detailed workflow for methylation analysis.

## Materials and methods

### Model species and study population

Spotted hyenas are keystone predators and scavengers which live in social groups called ‘clans’ that defend a territory^75^. Clans contain several generations of philopatric females, their offspring, and immigrant males^18^. Adult females are socially dominant over immigrant males, and there is a strict linear dominance hierarchy among adult females^14,18,75^. Hence, the lowest-ranking adult female is ranked above the highest-ranking immigrant male. All adult females reproduce, and births occur throughout the year. Litters contain one or two cubs, very rarely three^76^. Cubs shelter together in communal dens (Supplementary Method, Supplementary Fig. 1b) within clan territories for the first 12 months, and females typically only nurse their own offspring^19,65^. Cubs exclusively depend on highly nutritious milk during their first six months^19,77^ and are not weaned before 12–20 months of age^78^. Adulthood is set at 24 months^10,21,61^.

Our study area, the Serengeti National Park (NP) in Tanzania (Supplementary Fig. 1a) is characterised by the annual migration of large herds of wildebeest (*Connochaetes taurinus*), Thomson’s gazelles (*Eudorcas thomsonii*) and zebras (*Equus quagga*), the main prey of Serengeti hyenas^79^. From approximately early December, when the rains start, until the end of May, which is roughly the end of the wet season period, herds are in the east and southeast of the Serengeti NP. During the dry season (early June–end of November), herds move to areas in the west and north of the NP^79^. Hyenas regularly commute (potentially long distances) between their clan territories and areas where migratory herbivores are located^79,80^.

### Collection of field data and fecal samples in the Serengeti National Park

We collected data and fecal samples from individually known spotted hyenas between 2009 and 2019 in the context of an ongoing, individual-based long-term research project on three clans located at the centre of the Serengeti NP (Isiaka clan: monitored since 1987, Pool: since 1989, Mamba: since 1990; Supplementary Fig. 1a). During our routine field sessions, we observed hyenas in their clan territories at communal den sites during periods of several hours around dawn and dusk^19^. Study animals were habituated to the presence of observers in vehicles. During each field session we routinely scored the presence of all clan members within a radius of 100 m of the communal den(s). We individually recognised clan members by their spot patterns and cubs by ear notches, scars, or bald patches^81^. We estimated cub age ± 7 days using pelage characteristics and locomotion^81^. Cubs can be observed shortly after birth as they are nursed at the entrance to underground burrows^81^. As in previous studies, we defined cubs as individuals younger than one year (e.g., ref. ^10,16^). We routinely collected fresh fecal samples from clan members. Fecal samples were collected immediately after defecation and stored on cool packs in the field before freezing at our research station. In the current study, we only analyzed samples from females of both age classes (cubs and adults). Supplementary Methods provide further information about the Serengeti hyena study system.

### Collection of gut epithelium cells at the surface of feces, treatment, and storage

At the research station  in the Serengeti NP, we carefully removed the gut epithelium cells layer from the surface of fecal samples and mucus (that can contain gut epithelial cells, Supplementary Fig. 1b) with a scalpel that was cleaned in a flame before use. Samples were placed in either dimethyl sulfoxide (DMSO) or RNAlater (Thermo Fisher) and then stored at −10 °C or in liquid nitrogen before shipment to Germany, where they were stored at −80 °C. For the current study, we focused on females to avoid dealing with potential influences of sex on DNA methylation patterns and because we had more detailed information about the life histories of (philopatric) females than about males. We used 24 samples from female cubs and 18 from female adults. Adult samples originated from nine low and nine high-ranking females (Supplementary Table 1). Cub samples originated from cubs of 15 low-ranking mothers and 9 high-ranking mothers. Cubs were aged between two and nine months (Supplementary Tables 1 and 2).

### Determination of social rank and the two social classes

We determined the social rank of adult females using standard methods based on the observation of submissive acts in dyadic interactions recorded ad libitum and during focal observations^10,26^. We recorded submissive behaviors during dyadic interactions among adult females and constructed strictly linear dominance hierarchies for each clan (e.g., ref. ^10,15^). Dominance hierarchies were adjusted after each loss or recruitment of adult females and when dyadic interaction data revealed that an individual had increased or fallen in rank^10,15^. To compare ranks held by individuals within hierarchies containing different numbers of animals within and across clans, we calculated standardized ranks. This measure places the ranks within a given clan hierarchy evenly between the highest (standardised social rank: +1) and the lowest (standardised social rank: −1) rank^10,15^. We classified adult females with standardized ranks above and below the median rank as high-ranking and low-ranking, respectively. Adult females have high probabilities of staying within a social status class (yearly probabilities of staying high-ranking and low-ranking were equal to 0.94 and 0.97, respectively,^10^). Even so, we verified that the females from which we collected samples did not experience large variation in their standardized social ranks prior to sampling (Supplementary Fig. 2a). We attempted to select samples from females when they held a rank within the top or bottom of the hierarchy to maximise the chance of observing differences in DNA methylation patterns between high-ranking and low-ranking females. In our dataset, the 18 high-ranking females (9 cubs, 9 adults) had an average standardized social rank of 0.70 (ranging from +0.07 to +1), and the 24 low-ranking females (15 cubs, 9 adults) had an average standardised social rank of −0.56 (ranging from −0.07 to −1) (Supplementary Fig. 2a, Supplementary Table 2).

We assigned sampled cubs, i.e., individuals younger than one year, the social rank of their genetic mothers (e.g., ref. ^10^). During their long period of dependence on maternal milk, young spotted hyenas learn their social position, just below that held by their mothers in the clan hierarchy, by observing them interacting with other clan members and receiving social support from their mothers and closely related females^14,65^. In the population in the Maasai Mara National Reserve in Kenya, ~80% of cubs acquired the exact rank expected under such rules (maternal rank ‘inheritance’)^17^. Previous research in our study population and others show that maternal social status influences milk transfer and offspring growth rate during the first six months, survival to adulthood, and the age at first reproduction (e.g., ref. ^10,15,16,21,61^). Cubs of high-ranking mothers were also less likely to be infected with canine distemper virus and once infected more likely to survive the infection^10^. Thus, in both cubs and adults, social status influences life-history traits and measures of reproductive performance.

We conducted this study with research permits from the Tanzania Commission for Science and Technology and permission from the Tanzanian National Parks Authority and Tanzanian Wildlife Research Institute. All procedures were performed in accordance with the Leibniz Institute for Zoo and Wildlife Research Ethics Committee on Animal Welfare (permit number: 2017-11-02).

### DNA extraction and methylation capture

DNA from the gut epithelium cells was extracted using the NucleoSpin kit (Macherey und Nagel GmbH Co KG, Düren, Germany). DNA was sonicated to fragments of 300 to 400 bp length, with the Covaris SonoLab 7.1 M220. Fragment size was validated using a Tapestation D1000 and a ScreenTape kit (Agilent). Fragments <100 bp were removed with Ampure beads XP (Beckman Coulter GmbH, Krefeld, Germany). DNA concentrations were measured with the Qubit 2.0 Fluorometer (Invitrogen). The Methyl-binding domain (MBD) capture was performed using Invitrogen’s MethylMiner Methylated DNA Enrichment kit (Thermo Fisher, Waltham, USA), including the streptavidin-coated Dynabeads M-280 and the biotinylated mammalian methyl-binding protein domain 2 (MBD2). MBD2 specifically binds to methylated CpGs^82,83^, which are the main targets of methylation in the mammalian genomes, but not in bacterial DNA. Thus, the procedure enriches methylated and mammalian DNA simultaneously. Previously, this method had not been used for epithelium cells, but had been applied to enrich baboon DNA from feces^71^ (Supplementary Fig. 1c). After precipitation, DNA concentrations were again measured with the Qubit 2.0 Fluorometer (Invitrogen). Internal controls were applied to ensure sufficient capture of methylated *versus* non-methylated DNA.

### Library preparation and high-throughput sequencing

Illumina sequencing libraries with the methylated MBD-enriched DNA were prepared using the NEBNext® Ultra™ II DNA Library Prep Kit (Frankfurt, Germany) following the manufacturer’s instructions with some modifications. End-repair and adaptor ligation followed the protocol using the methylated adaptor #E7536AA. Size selection was performed with SPRI beads (AMPure Beads XP, Beckman Coulter GmbH, Krefeld, Germany). DNA samples from spotted hyenas were prepared with a previously established protocol that consisted of a first right-side size selection step with 0.6×, followed by left-side size selection with a ratio of 1.8× of the supernatant fraction. Samples were pooled and sequenced on a HiSeq2000 machine at Macrogen, Inc. (Seoul, Südkorea) using the kit HiSeqX 150 bp PE sequencing run, resulting in up to 300 bp long sequencing reads.

### Bioinformatic data pre-processing, quality control, and mapping

Raw sequencing data were then converted by Macrogen into FASTQ files using the conversion software bcl2fastq^84^. Adaptors were clipped using the software cutadapt (v2.4,^85^), and using Trimmomatic (v.0.39,^86^) adaptor-clipped reads were then quality-filtered through a sliding window approach of 10-base wide window for an average quality of at least Q20 within the window. Reads shorter than 20 bp were discarded (Supplementary Table 1). Quality-filtered (clean) reads were mapped to the novel spotted hyena genome assembly available at the Genome Warehouse in the National Genomics Data Centre (http://bigd.big.ac.cn/gwh/) under BioProject accession code: PRJCA004316 (accession code: GWHAZPN00000000) using the *bowtie 2* mapper (v3.5.1,^87^). Data were sorted and duplicates removed using *SAMtools* (v1.3,^88^).

### Reference sequence validation and custom annotation

Three spotted hyena genomes were recently sequenced and published^49,89^, and DNAZoo https://www.dnazoo.org/assemblies/Crocuta_crocuta). We compared reference genomes and chose the one giving the best results with BUSCO (Supplementary Table 5), and after mapping with different input datasets (Supplementary Table 6). The reference genome provided by University of Potsdam^49^ revealed the best mapping results and was further applied^49^. The genome sequence is a high-quality long-read genome assembly generated by a combination of PacBio long reads and short-read sequences of the spotted hyena, with a contig N50 length of ~13.75 Mb. It is accessible to the Genome Warehouse in the National Genomics Data Centre (http://bigd.big.ac.cn/gwh/) under BioProject accession code: PRJCA004316 (accession code: GWHAZPN00000000) and is thus publicly available.

The annotation published together with the spotted hyena genome^49^ lacked several annotations important for gene regulation and epigenetics. We performed the custom annotation file in which we incorporated relevant positions for epigenetic regulation (i.e., gene annotations, Transcription Start Sites (TSS), promoters). In a first step, we mapped the annotation of the recently published striped hyena (*Hyaena hyaena)* annotation (RefSeq Assembly ID: GCF_003009895.1) to the spotted hyena genome^49^ as closest relative using the Liftoff software (v1.6.1,^90^). This provided the annotation information “exon”, “gene”, “transcript”, “start_codon”, “CDS”, “stop_codon”. In a second step, we annotated transcription start sites (TSS) as the first position of the first exon of a gene, and the “promoter” regions as 2000 bp upstream the “TSS”. The annotation file includes the following functional regions (features): “promoter”, “TSS”, “exon”, “gene”, “transcript”, “start_codon”, “CDS” and “stop_codon”. Exons for mitochondrial genes were added. Introns were calculated by subtraction of gene minus exon. Annotation was done with bash commands and R v.3.6^91^, and are available  in Dryad (10.5061/dryad.m0cfxpp9b).

### RNA-Seq

RNA-Seq was performed on 16 samples of adults (10 high-ranking and 6 low-ranking females). Total RNA was measured with Qubit, and RIN values were checked by TapeStation. Samples were provided to the Genomic Centre at Max Delbrück Centre for Molecular Medicine, Berlin, Germany. There, Illumina sequencing libraries were prepared using a combination of the NEBNext Ultra II Directional RNA Library Prep Kit, the NEBNext Total RNA kit for indexing, and the NEBNext Multiplex Oligos for Illumina with index primers (Set 1 or 2 or 3 or 4). Sequencing was done with the NEBNext total RNA, HiSeq4000 on 2 lanes, PE150 (Frankfurt, Germany), resulting in up to 300 bp long sequencing reads. Bioinformatic computation was described above, including that total RNA samples were filtered for the presence of rRNA sequences using SortMeRNA (v4.3.3^92^), before the quality filter step with Trimmomatic. For mapping, we used STAR (v2.7.9a^93^).

### Statistics and reproducibility

#### Methylation analysis

The MBD protein binds to methylated DNA fragments in genomic regions. More methylation in this region results in higher numbers of sequenced reads in this region and thus higher read counts (more read counts = more methylated, less reads counts = less methylated).

All analyses were performed using R version 4.2.1^91^. Data preparation was done using the Bioconductor package MEDIPS^94^, and modelling was performed using the EdgeR package^95^. We first created genomic windows of 300 bp and normalised libraries using the method trimmed mean of M-values (TMM) (edgeR:normLibSizes), which normalises the library sizes to minimise the log fold-changes between the samples. Second, windows were filtered for a minimal total coverage of 10 reads per position and a minimum of 10 different individuals with positive read count values. These windows represented our regions of interest, unless stated otherwise. Third, we fitted a negative binomial model using the edgeR::glmFit() function with age class (cub, adult) and social status class (high-ranking, low-ranking) as fixed effects. The variance was estimated using the weighted likelihood empirical Bayes method (edgeR::EstimateDisp()). *P* values were computed with Likelihood Ratio Tests (edgeR::glmLRT), and false discovery rate (FDR) controlled with the Benjamini-Hochberg procedure^96^. Windows with a FDR ≤ 0.05 were considered significantly different, and adjacent significant windows were merged to create DMRs. In genomic regions which are differentially methylated (DMRs), the variation within groups (high-ranking or low-ranking) is lower than between groups. Finally, differentially methylated regions (DMRs) were annotated to their functional genomic region, using the novel custom-made annotation, including promoters, exons, introns, splice sites, 3′- and 5′-untranslated regions (3′UTRs, and 5′UTRs) of genes and intergenic regions. For four uncharacterised genes (with LOC ids), we identified gene names using the NCBI database search function. The STRING online tool^97,98^; https://string-db.org, accessed on 03 January 2023; *Felis catus* genome used as reference) was used for network analysis with default settings and robust differentially methylated protein-encoding genes for detecting protein–protein interactions.

#### Subsampling procedure

To assess the coherence of the direction of the effect of rankDMRs found, we randomly assigned the 42 samples to six subgroups, containing seven samples each. We then generated subsampling datasets (i.e., “subsets”), consisting of five subgroups. In total, we had six subsets, containing 35 samples each, which is representative of sample size in other studies (e.g., ref. ^44^). The methylation analysis was rerun per subset. The subsampling was done randomly, with the only condition that each subgroup contained at least one instance of high-ranking and low-ranking and one instance of young and adult. We retained the effect size and *p* values of the rankDMRs found in the full datasets. RankDMRs with a *p* value ≤ 0.05 in each subset and with a coherent effect direction across the subsets (e.g., always hypermethylated) were considered robust.

#### Correlation of effect for age classes

To address the possibility that one age class drives the results, we analyzed the effect of rank on read counts in cubs and adults separately in the 179 rankDMRs. We fitted two negative binomial models (one for adults and one for cubs) following the same procedure as described above (see Methylation analysis). The differences being that: 1. Only social status class (high-ranking, low-ranking) was included as fixed effect, and 2. Only the 321 windows composing the rankDMRs were analyzed. We then computed Pearson’s product-moment coefficient of correlation between the effect (logFC) in cubs and adults. The correlation was computed using the stats::cor.test() function. Finally, we repeated this procedure 1000 times on random sub-samples of 321 windows each. We thus obtained a distribution of coefficients of correlation with which we computed the empirical *p* value.

#### Discriminative power analysis

To assess the discriminative power of the observed rankDMRs and compare it to other measures such as the mean methylation, we trained four random forest classifiers on the subsets of interest (total rankDMRs, selected rankDMRs, excluded rankDMRs, mean DNA methylation). A random forest classifier is a learning procedure that operates by creating decision trees using a subset of the variables and a subset of the samples. The samples not used to build a tree (out-of-bag-samples) are used as a test-set for the classification outcome of this tree. The average classification error on the out-of-bag-samples (oob-error) provides an estimate of the general error rate. We use oob-error as a proxy for the discriminative power of a group of rankDMRs. While oob-errors are insightful in a comparative setting, their absolute value should be taken with caution as we used the same dataset to select the rankDMR and to train the random forest classifier.

Random forest provides a straightforward tool to manipulate large numbers of covariates and as such represent a suitable predictive tool for our purpose. We used the read Count Per Million (CPM, adjusted for library size using TMM) of the rankDMRs of interest as covariates for the random forest classifiers. We fitted the random forest with the ranger R-package^99^ using 2000 trees, keeping the other parameters at their default values, without trying to maximise the predictive power by adjusting the meta-parameter.

The formulae of the classifiers were as follows:

$${{{{{\rm{RF}}}}}}1{{{{{\rm{total}}}}}}{{\_}}{{{{{\rm{rankDMRs}}}}}}\!: \ \scriptstyle{{{{{{\rm{rank}}}}}} \, \sim {{{{{\rm{age}}}}}}\,+\,{{{{{\rm{CPM}}}}}}\,{{{{{\rm{in}}}}}}\,{{{{{\rm{DMR}}}}}}1\, + \,{{{{{\rm{CPM}}}}}}\,{{{{{\rm{in}}}}}}\,{{{{{\rm{DMR}}}}}}2\ldots}$$

$${{{{{\rm{RF}}}}}}2{{{{{\rm{selected}}}}}}{{\_}}{{{{{\rm{rankDMRs}}}}}}\!: \ \scriptstyle{{{{{{\rm{rank}}}}}} \sim {{{{{\rm{age}}}}}}\,+\,{{{{{\rm{CPM}}}}}}\,{{{{{\rm{in}}}}}}\,{{{{{\rm{selected}}}}}}\,{{{{{\rm{DMR}}}}}}1\, +\,{{{{{\rm{CPM}}}}}}\,{{{{{\rm{in}}}}}}\,{{{{{\rm{selected}}}}}}\,{{{{{\rm{DMR}}}}}}2\ldots}$$

$${{{{{\rm{RF}}}}}}3{{{{{\rm{excluded}}}}}}{{\_}}\,{{{{{\rm{rankDMRs}}}}}}\!: \ \scriptstyle{{{{{{\rm{rank}}}}}} \sim \,{{{{{\rm{age}}}}}}\,+\,{{{{{\rm{CPM}}}}}}\,{{{{{\rm{in}}}}}}\,{{{{{\rm{excluded}}}}}}\,{{{{{\rm{DMR}}}}}}1\, + \,{{{{{\rm{CPM}}}}}}\,{{{{{\rm{in}}}}}}\,{{{{{\rm{excluded}}}}}}\,{{{{{\rm{DMR}}}}}}2\ldots}$$

$${{{{{\rm{RF}}}}}}4{{{{{\rm{mean}}}}}}{{\_}}{{{{{\rm{DNA methylation}}}}}}\! : \ \scriptstyle{{{{{{\rm{rank}}}}}} \sim {{{{{\rm{age}}}}}}+{{{{{\rm{Mean}}}}}}\,{{{{{\rm{DNA methylation}}}}}}}$$

In addition, we controlled for the different number of variables used to train the RFs by training a new set of four RFs on randomly selected locations (between 1 and 179) using the same number of variables as for the four RFs in the main analysis (Supplementary Table 4). This control showed that no model performed better than would have been by chance, irrespective of the number of variables used to train the model.

### Mitochondrial and nuclear DNA and RNA comparison

We compared mitochondrial and nuclear gene expression (RNA-Seq) and DNA methylation (MBD-Seq). For the analysis of gene expression, we used RNA from samples from 16 different adult females (10 high-ranking and 6 low-ranking). Expression data were processed using the same pipeline as for the DNA methylation analysis. The only difference is that the region of interest consisted of transcript length per gene instead of genomic windows of 300 bp. We then computed the mean Count Per Million (cpm; function edgeR::CPM()) for all transcripts and all individuals in mitochondrial genes (*n* = 8) and nuclear genes (*n* = 7056). We did the same for the DNA methylation using the mean cpm across all windows overlapping with mitochondrial and nuclear DNA. As the groups were independent in the comparison between high-ranking and low-ranking hyenas, we computed the *p* values using the Wilcoxon rank-sum test. However, the samples being dependent for the comparison between the mitochondrial vs nuclear genes we computed the *p* value using the Wilcoxon signed rank test using the coin R package^100^.

### Reporting summary

Further information on research design is available in the Nature Portfolio Reporting Summary linked to this article.

### Supplementary information

Supplementary Material

Reporting Summary

## Data Availability

The MBD-Seq data are uploaded to NCBI BioProject ID PRJNA1036526, including reference genome “crocuta.fasta”. The statistical analysis is available as a R-package Weyrich23 at https://github.com/vullioud/Weyrich23. Novel annotation file and read count tables are available here 10.5061/dryad.m0cfxpp9b^101^. Supplementary Methods and Supplementary Results provide further information.
