# Supplementary material for: Epigenetic signatures of social status in wild female spotted hyenas (Crocuta crocuta)
Source: Commun Biol. 2024 Mar 28;7:313. doi: 10.1038/s42003-024-05926-y (PMC10978994; doi:10.1038/s42003-024-05926-y)
Supplement: Supplementary file 1 — Supplementary Material [file 42003_2024_5926_MOESM1_ESM.pdf]

Epigenetic signatures of social status in wild female free-ranging spotted hyenas (Crocuta crocuta)

Colin Vullioud, Sarah Benhaiem, Dorina Meneghini, Moshe Szyf, Yong Shao, Heribert Hofer, Marion L. East, Jörns Fickel, Alexandra Weyrich

Supplementary Material

Content:

I. **Supplementary Methods**
II. **Supplementary Results**
III. **References**

I. Supplementary Methods

Social rank stability in female spotted hyenas

In the Serengeti National Park, in Tanzania, East Africa (Supplementary Figure 1a), we observed individual spotted hyenas in their clan territories (Supplementary Figure 1b) and collected faecal samples (gut epithelium cells and mucus) non-invasively (Supplementary Figure 1c) from known animals, in the context of a long-term project. We observed the outcomes of dyadic social interactions and used standard methods to calculate standardised social ranks which place individual social ranks within a given clan hierarchy evenly between the highest (standardised social rank: +1) and the lowest (standardised social rank: −1) rank. For cubs, we assigned the standardised social rank of their genetic mothers. Here we defined adult females with standardised social ranks above and below the median social rank as high-ranking and low-ranking, respectively - these are the two main social classes considered in this study. Spotted hyenas live in complex fission-fusion societies structured by linear dominance hierarchies and in our study population, clans may contain up to 50 adult females, 50 cubs (<1 year) and subadults (1-2 years old), 20 reproductively active natal males and 30 adult immigrant males. We used faecal samples from females in three clans in the centre of the Park (Isiaka, Mamba and Pool clans, Supplementary Figure 1a). We conducted standard field observations at clan communal dens, the social centres of the clan where individuals gather and cubs are kept in burrows (Supplementary Figure 1b). We collected fresh faecal samples (Supplementary Figure 1c) when clan members defecated near the clan communal dens. We carefully removed gut epithelial cells and mucus from each faecal sample. We then

isolated the DNA and enriched this material for mammalian methylated DNA, using MBD2 for capture (Supplementary Figure 1c).

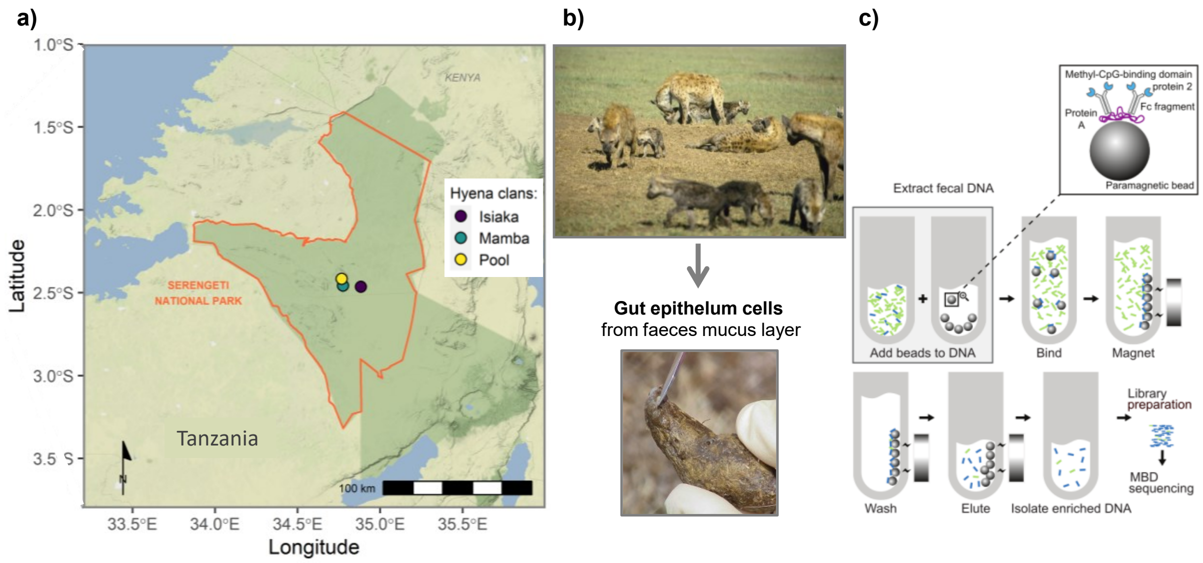

Supplementary Figure 1. Graphical abstract of study design. a) Locations of the three spotted hyena clans ('Isiaka', 'Mamba', 'Pool') in the Serengeti National Park in Tanzania, Africa (Map by M. Gicquel). b) Close observations of spotted hyenas at the clan communal den in their territory (credit: M.L East) and collection of gut epithelium cells as sample material to extract DNA for methylation analysis. c) Illustration of the method using MBD-Seq of DNA from faecal epithelium cells. Spotted hyena gut epithelium cells were used for DNA extraction. After DNA fragmentation, the MBD2 protein coupled with a biotin molecule was added to the DNA, where it specifically binds to methylated CpG positions (mCpG) in the DNA fragments, and thus to methylated mammalian DNA (blue fragments), rather than bacteria DNA (green fragments). Methylated DNA bound to MBD2 bound to streptavidin-coated paramagnetic beads was then enriched using a magnetic rack. Only methylated DNA was used for library preparation followed by high-throughput sequencing (MBD-Seq) (Figure adapted from[1], done for faeces samples in baboons).

In this study population as in others, dominance hierarchies in clans are generally stable and reversals are rare[2–4]. With age, females tend to experience a decrease in their social rank, due to demographic changes, a potential decline in their body condition or social rank reversals in which mothers are outcompeted by their adult daughters, as in other nepotistic societies[5,6]. We verified that the females we used samples from did not experience major changes in their social rank prior to sampling (Supplementary Figure 2a).

II. Supplementary Results

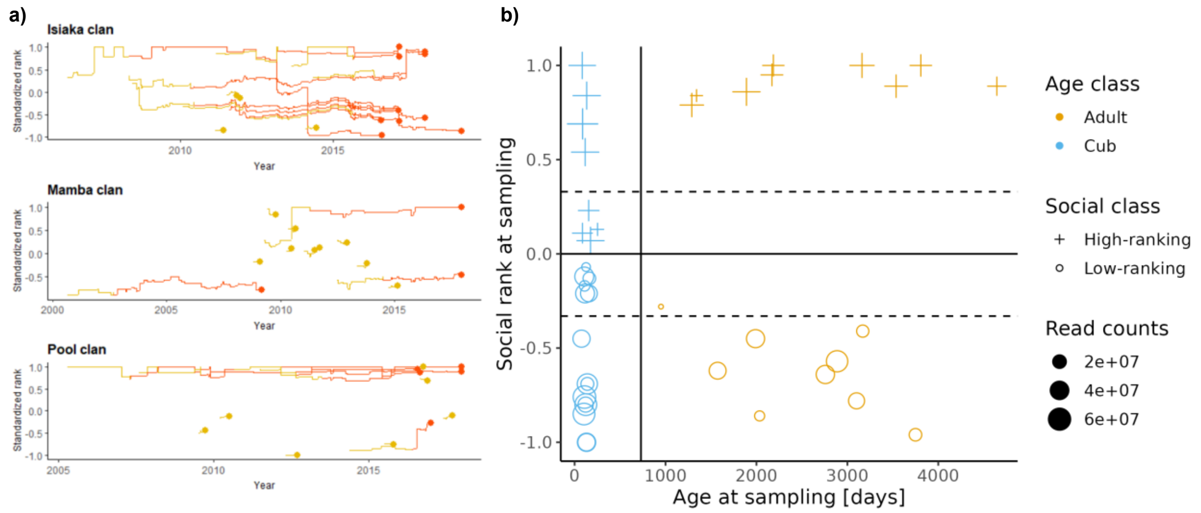

Supplementary Figure 2. Standardised social ranks of sampled female spotted hyenas as a function of time. a) Variation in standardised social ranks of each sampled individual, prior to the sample collection in both cubs (n= 24) and adults (n= 18). Yellow dots indicate the dates of sampling for cubs and red dots the dates of sampling for adults. Yellow lines represent the standardised social rank of cubs (i.e. the social rank of their mothers) and red lines the standardised social rank of adults. b) Social rank at sampling date as a function of age at sampling and library size. Adjusted read counts per sampled hyena as a function of its age (in days) and its standardised social rank (HR: high-ranking (cross), LR: low-ranking (circle)). The number of reads per library is illustrated by the size of crosses and circles.

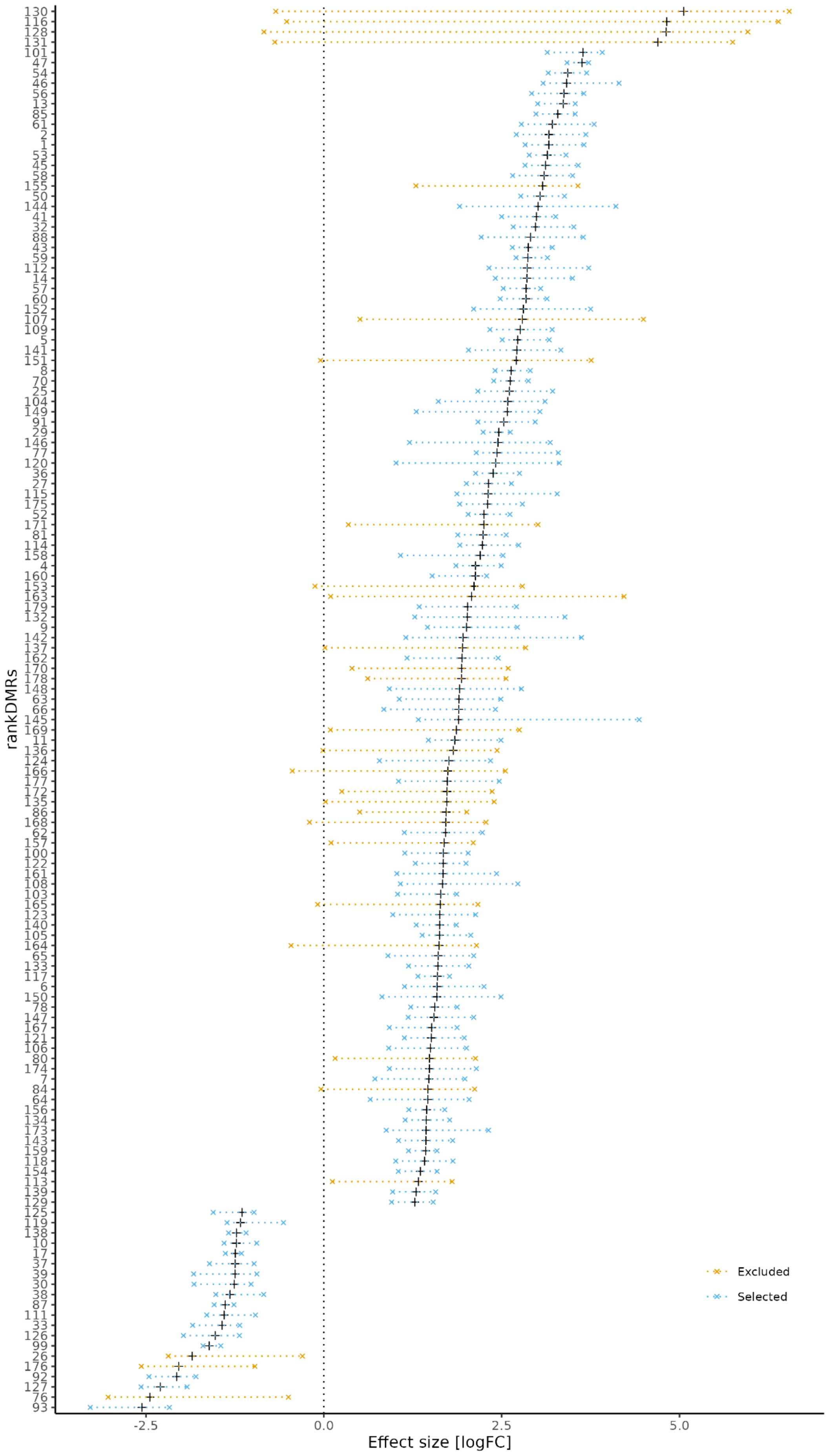
rankDMRs
Effect size [logFC]
-2.5
0.0
2.5
5.0
Excluded
Selected

Supplementary Figure 3. DNA methylation differences between high- and low-ranking female spotted hyenas in intergenic regions according to their fold-change, hyper- and hypomethylation and effect range. The black cross is the observed logFC value in the full dataset. The x-symbols and the dashed lines mark the logFC range across the subsets. Blue dashed lines indicate that the direction of the effect (hypermethylation *vs.* hypomethylation) across the subsets was coherent (selected rankDMRs), and the orange dashed lines indicates that the effect was incoherent (excluded rankDMRs). See Figure 2 of the article for equivalent results in intragenic regions.

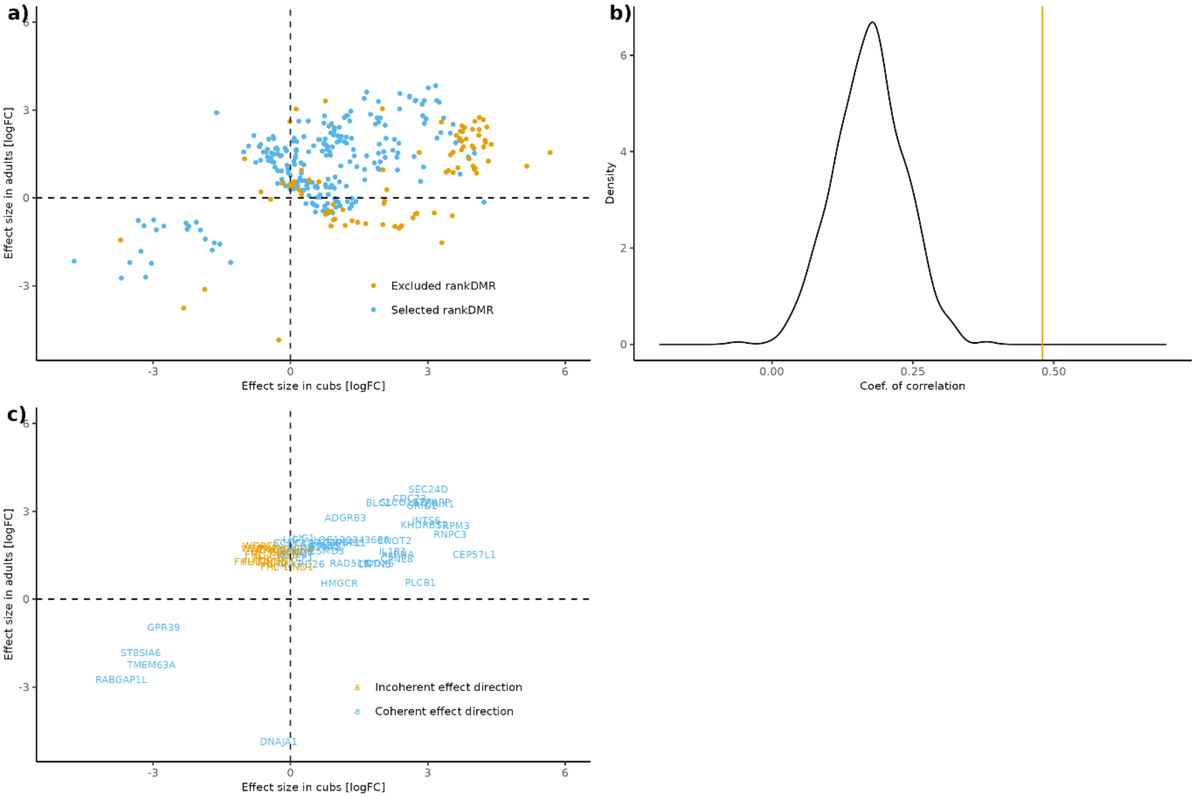

Supplementary Figure 4. Correlation between DNA methylation differences between high- and low-ranking female hyenas in cubs and adults. a) LogFC in rankDMRs (n= 179, $n_{windows}$= 321) in cubs (n= 24) and adults (n= 18), while analysing the two subgroups separately. b) Density distribution of the Pearson's coefficient of correlation between the logFC observed while fitting the model separately for cubs and adults. The distribution was obtained by bootstrapping 1000 coefficient of Pearson correlations between the logFC observed in cubs and adults in 321 randomly selected windows (see method above). The blue line represents the coefficient of correlation measured for the selected rankDMRs and the yellow line for the total rankDMRs. c) LogFC in rankDMRs (n= 42, $n_{windows}$= 52) overlapping genes in cubs and adults, while analysing the two subgroups separately.

Supplementary Table 1: Individual age, social status, standardised social rank at sampling date, and sequencing results of MBD captured methyl DNA derived from gut epithelium cells from female cubs and adults.

No.	Clan	Age at sampling [days]	Social status	Social rank	Age class	Raw reads	Clean paired reads	Read length [bp]	%GC	Mapping efficiency [%]
ID1	I	103	low-ranking	-0.85	cub	50315708	49292951	20-151	45	83.09
ID2	I	162	low-ranking	-0.13	cub	15841755	15223009	20-151	43	67.69
ID3	I	126	low-ranking	-0.07	cub	9451983	8662306	20-151	42	30.99
ID4	I	123	low-ranking	-0.8	cub	70267493	68778916	20-151	44	60.86
ID5	I	121	low-ranking	-0.8	cub	37268057	36121697	20-151	45	46.61
ID6	M	110	low-ranking	-0.17	cub	31246715	30629453	20-151	53	20.32
ID7	M	133	high-ranking	0.84	cub	42228373	41202865	20-151	48	77.13
ID8	M	86	high-ranking	0.11	cub	46551045	45664979	20-151	46	28.04
ID9	M	116	high-ranking	0.54	cub	42147876	41553124	20-151	49	70.76
ID10	M	252	high-ranking	0.13	cub	16016908	15021595	20-151	47	16.82
ID11	M	175	high-ranking	0.07	cub	36672196	36353514	20-151	49	93.96
ID12	M	155	high-ranking	0.23	cub	16929464	16134091	20-151	56	77.36
ID13	M	157	low-ranking	-0.21	cub	43338426	42470708	20-151	49	42.99
ID14	M	140	low-ranking	-0.69	cub	11160125	10562029	20-151	55	75.77
ID15	M	140	low-ranking	-0.69	cub	44560221	44163448	20-151	52	76.84
ID16	P	76	low-ranking	-0.45	cub	44291341	41251714	20-151	47	47.99
ID17	P	106	low-ranking	-0.12	cub	38321090	36771768	20-151	45	75.60
ID18	P	131	low-ranking	-1	cub	50671362	48814934	20-151	52	59.51
ID19	P	131	low-ranking	-1	cub	55274104	54681890	20-151	45	43.66
ID20	P	108	low-ranking	-0.76	cub	50974534	49491625	20-151	50	93.23
ID21	P	115	low-ranking	-0.21	cub	48025226	47403408	20-151	45	61.07
ID22	P	84	high-ranking	1	cub	34302539	32348837	20-151	46	86.07
ID23	P	87	high-ranking	0.69	cub	44102295	42108643	20-151	48	90.42
ID24	P	88	high-ranking	0.69	cub	49768566	49091667	20-151	50	89.40
ID25	I	1289	high-ranking	0.79	adult	31008389	30606990	20-151	45	85.00
ID26	I	1341	high-ranking	0.84	adult	48725321	45551372	20-151	47	77.99
ID27	P	1890	high-ranking	0.86	adult	94330915	92630770	20-151	47	51.74
ID28	P	2170	high-ranking	0.95	adult	58026316	56802549	20-151	46	44.17
ID29	I	2186	high-ranking	1	adult	48119257	46766504	20-151	41	43.44
ID30	M	3161	high-ranking	1	adult	37193335	36762499	20-151	47	80.20
ID31	I	3536	high-ranking	0.89	adult	35813646	35196852	20-151	50	74.19
ID32	P	3808	high-ranking	1	adult	114864861	113255861	20-151	47	19.65
ID33	P	4644	high-ranking	0.89	adult	57872668	56439111	20-151	42	24.01
ID34	P	950	low-ranking	-0.28	adult	51189360	46876621	20-151	43	56.59
ID35	I	1573	low-ranking	-0.62	adult	50925578	50509114	20-151	56	91.54
ID36	M	1990	low-ranking	-0.45	adult	57294827	56355236	20-151	43	58.10
ID37	I	2035	low-ranking	-0.86	adult	47362353	45958509	20-151	41	14.78
ID38	I	2758	low-ranking	-0.64	adult	42629659	41796235	20-151	47	85.27
ID39	M	3101	low-ranking	-0.78	adult	50713223	50150777	20-151	40	49.38
ID40	I	3169	low-ranking	-0.41	adult	84305138	82853137	20-151	44	15.49
ID41	I	3749	low-ranking	-0.96	adult	43120760	41900788	20-151	40	28.28
ID42	I	2887	low-ranking	-0.57	adult	53395261	52843127	20-151	45	15.03

Raw sequencing reads were computed bioinformatically to clean reads. Social rank is standardised.

Supplementary Table 2: Sample collection with mean age and rank, data classification and mean absolute values within each group.

Age	rank	n	mean age (days)	mean rank (std rank)
cub	high-ranking	9	132	0.48
cub	low-ranking	15	123	-0.53
adult	high-ranking	9	2669	0.91
adult	low-ranking	9	2468	-0.62

Supplementary Table 3: RankDMR length above 300 bp overlapping with genes

**RankDMR length [bp]**		
**1500 bp**	**900 bp**	**600 bp**
FHL-1	EPH6A	CEP57L1
ND-1	ND4	LIG1
ND-5	WDPCP	

Supplementary Table 4: Range of out-of-bag-samples (oob) error for 1000 repetition of Random Forest trained on random position.

**RF training variable**	**oob-error range**
Random-total (n= 321)	33.3 – 64.4%
Random robust (n= 233)	35.7 – 66.7%
Random non-robust (n= 88)	28.6 – 69.0%
Random mean-methylation (n= 1)	21-4 – 73.8%

Supplementary Table 5: Reference genomes comparison by BUSCO

	Run 1) University of Potsdam Genome	Run 2) BGI "new" NCBI	Run 3) DNA Zoo
BUSCO notation for file	crocuta.fasta	GCA_008692635.1_BGI_CrCroc_1.0_genomic.fna	Crocuta_crocuta_HiC.fasta
	**Results**		
Complete BUSCOs (C)	6382	5901	5868
Complete and single-copy BUSCOs (S)	6380	5897	5859
Complete and duplicated BUSCOs (D)	2	4	9
Fragmented BUSCOs (F)	592	1013	712
Missing BUSCOs (M)	2252	2312	2646
Total BUSCO groups searched	9226	9226	9226

For an inter-genome comparison, we ran BUSCO v4.1.3 in mode 'genome' on dataset 'mammalia_odb10' (eukaryota, 2020-08-05) consisting of 9226 total BUSCO groups and compared the output. The genome by Shao et al. 2022 (provided to us by the University of Potsdam before publication) resulted in higher number of "complete and single-copy BUSCOs genes" in comparison to BGI and DNA Zoo reference genomes. Because DNA Zoo had lowest quality, we did additional quality test focussing on the two genomes with similar and best results: BGI and University Potsdam[7].

Supplementary Table 6: Mapping for reference genomes comparison

Sample_id_Genome for Mapping	Sequence pairs analysed in total	Number of paired-end alignments with a unique best hit	Mapping efficiency	Total number of C's analysed	Total methylated C's in CpG context
**Mapping of sample 565**					
**EpiRank_565_UniPotsdam genome**	**376311349**	**21613851**	**5.7%**	**866085631**	**33426685**
EpiRank_565_BGI genome	376311349	21214330	5.6%	847429266	32049988
**Mapping of sample 405**					
**EpiRank_405_UniPotsdam genome**	**361387186**	**114730919**	**31.7%**	**4529492533**	**188581915**
EpiRank_405_BGI genome	361387186	112430914	31.1%	4424610834	181204398

In a second quality check, we performed mapping to the two reference genomes University of Potsdam Genome (UniPotsdam genome) and BGI genome, using clean reads of two samples (EpiRank_565 and EpiRank_405) which were sequenced by whole genome bisulfite (WGS) (but not further used for MBD-Seq). We mapped the full length of reads of both samples to both genomes, respectively, using the Bismark Bisulfite Read Mapper and Methylation Caller[8]. We evaluated sequence pairs analysed in total, the number of paired-end alignments with a unique hit, the total number of mapped reads, the mapping efficiency, the total number of C`s analysed and the total methylated C`s in CpG context. Overall, the reference provided to us by the University Potsdam published by Shao et al. [7] provided the best mapping results.

III. Supplementary References

1. Chiou, K. L. & Bergey, C. M. Methylation-based enrichment facilitates low-cost, noninvasive genomic scale sequencing of populations from feces. *Sci Rep* **8**, 1975 (2018).
2. Vullioud, C. *et al.* Social support drives female dominance in the spotted hyaena. *Nat Ecol Evol* **3**, 71–76 (2019).
3. Marescot, L. *et al.* Social status mediates the fitness costs of infection with canine distemper virus in Serengeti spotted hyenas. *Funct Ecol* **32**, 1237–1250 (2018).
4. Strauss, E. D., Shizuka, D. & Holekamp, K. E. Juvenile rank acquisition is associated with fitness independent of adult rank. *Proceedings of the Royal Society B: Biological Sciences* **287**, 20192969 (2020).
5. Combes, S. L. & Altmann, J. Status change during adulthood: life-history by-product or kin selection based on reproductive value? *Proc Biol Sci* **268**, 1367–1373 (2001).
6. Lea, A. J., Learn, N. H., Theus, M. J., Altmann, J. & Alberts, S. C. Complex sources of variance in female dominance rank in a nepotistic society. *Anim Behav* **94**, 87–99 (2014).

7. Shao, Y. *et al.* Long-Read Genome Sequencing Provides Molecular Insights into Scavenging and Societal Complexity in Spotted Hyena Crocuta crocuta. *Mol Biol Evol* **39**, msac011 (2022).
8. Krueger, F. & Andrews, S. R. Bismark: a flexible aligner and methylation caller for Bisulfite-Seq applications. *Bioinformatics* **27**, 1571–1572 (2011).
