# Supplementary material for: Epigenetic signatures of social status in wild female spotted hyenas (Crocuta crocuta)
Source: Commun Biol. 2024 Mar 28;7:313. doi: 10.1038/s42003-024-05926-y (PMC10978994; doi:10.1038/s42003-024-05926-y)
Supplement: Supplementary file 2 — Reporting Summary [file 42003_2024_5926_MOESM2_ESM.pdf]

nature portfolio

Corresponding author(s): Alexandra Weyrich

Last updated by author(s): 31/01/24

Reporting Summary

Nature Portfolio wishes to improve the reproducibility of the work that we publish. This form provides structure for consistency and transparency in reporting. For further information on Nature Portfolio policies, see our Editorial Policies and the Editorial Policy Checklist.

Statistics

For all statistical analyses, confirm that the following items are present in the figure legend, table legend, main text, or Methods section.

n/a	Confirmed	
☐	☒	The exact sample size (*n*) for each experimental group/condition, given as a discrete number and unit of measurement
☐	☒	A statement on whether measurements were taken from distinct samples or whether the same sample was measured repeatedly
☐	☒	The statistical test(s) used AND whether they are one- or two-sided<br>*Only common tests should be described solely by name; describe more complex techniques in the Methods section.*
☐	☒	A description of all covariates tested
☐	☒	A description of any assumptions or corrections, such as tests of normality and adjustment for multiple comparisons
☐	☒	A full description of the statistical parameters including central tendency (e.g. means) or other basic estimates (e.g. regression coefficient) AND variation (e.g. standard deviation) or associated estimates of uncertainty (e.g. confidence intervals)
☐	☒	For null hypothesis testing, the test statistic (e.g. *F*, *t*, *r*) with confidence intervals, effect sizes, degrees of freedom and *P* value noted<br>*Give P values as exact values whenever suitable.*
☒	☐	For Bayesian analysis, information on the choice of priors and Markov chain Monte Carlo settings
☐	☒	For hierarchical and complex designs, identification of the appropriate level for tests and full reporting of outcomes
☐	☒	Estimates of effect sizes (e.g. Cohen's *d*, Pearson's *r*), indicating how they were calculated

Our web collection on statistics for biologists contains articles on many of the points above.

Software and code

Policy information about availability of computer code

Data collection	We collected faecal gut epithelium cells, extracted DNA and RNA and performed next generation sequencing (Illumina). Sequencing data were used for bioinformatic processing, read mapping and data analysis.
Data analysis	All analyses were performed using R version 4.2.1 (R Core Team, 2022).

For manuscripts utilizing custom algorithms or software that are central to the research but not yet described in published literature, software must be made available to editors and reviewers. We strongly encourage code deposition in a community repository (e.g. GitHub). See the Nature Portfolio guidelines for submitting code & software for further information.

Data

Policy information about availability of data

All manuscripts must include a data availability statement. This statement should provide the following information, where applicable:

- Accession codes, unique identifiers, or web links for publicly available datasets
- A description of any restrictions on data availability
- For clinical datasets or third party data, please ensure that the statement adheres to our policy

DNA methylation data were uploaded to NCBI BioProject ID PRJNA1036526, including reference genome "crocuta.fasta". The statistical analysis is available as a R-package “Weyrich23” at https://github.com/vullioud/Weyrich23. Novel annotation file and read count tables are available here https://doi.org/10.5061/dryad.m0cfxpp9b.

Research involving human participants, their data, or biological material

Policy information about studies with human participants or human data. See also policy information about sex, gender (identity/presentation), and sexual orientation and race, ethnicity and racism.

Reporting on sex and gender	*Use the terms sex (biological attribute) and gender (shaped by social and cultural circumstances) carefully in order to avoid confusing both terms. Indicate if findings apply to only one sex or gender; describe whether sex and gender were considered in study design; whether sex and/or gender was determined based on self-reporting or assigned and methods used. Provide in the source data disaggregated sex and gender data, where this information has been collected, and if consent has been obtained for sharing of individual-level data; provide overall numbers in this Reporting Summary. Please state if this information has not been collected. Report sex- and gender-based analyses where performed, justify reasons for lack of sex- and gender-based analysis.*
Reporting on race, ethnicity, or other socially relevant groupings	*Please specify the socially constructed or socially relevant categorization variable(s) used in your manuscript and explain why they were used. Please note that such variables should not be used as proxies for other socially constructed/relevant variables (for example, race or ethnicity should not be used as a proxy for socioeconomic status). Provide clear definitions of the relevant terms used, how they were provided (by the participants/respondents, the researchers, or third parties), and the method(s) used to classify people into the different categories (e.g. self-report, census or administrative data, social media data, etc.) Please provide details about how you controlled for confounding variables in your analyses.*
Population characteristics	*Describe the covariate-relevant population characteristics of the human research participants (e.g. age, genotypic information, past and current diagnosis and treatment categories). If you filled out the behavioural & social sciences study design questions and have nothing to add here, write "See above."*
Recruitment	*Describe how participants were recruited. Outline any potential self-selection bias or other biases that may be present and how these are likely to impact results.*
Ethics oversight	*Identify the organization(s) that approved the study protocol.*

Note that full information on the approval of the study protocol must also be provided in the manuscript.

Field-specific reporting

Please select the one below that is the best fit for your research. If you are not sure, read the appropriate sections before making your selection.

☐ Life sciences ☒ Behavioural & social sciences ☐ Ecological, evolutionary & environmental sciences

For a reference copy of the document with all sections, see nature.com/documents/nr-reporting-summary-flat.pdf

Behavioural & social sciences study design

All studies must disclose on these points even when the disclosure is negative.

Study description	In the current study, we tested whether social status is reflected by specific DNA methylation profiles in cubs and adult in free-ranging spotted hyenas (Crocuta crocuta). This social carnivore is living in groups structured by a linear dominance hierarchy, in which female social status influences access to resources, health, reproductive performance and survival, and movement patterns. Using non-invasively collected faecal gut epithelium cells from 42 female spotted hyenas (cubs and adults) with known social status in the Serengeti National Park, Tanzania, we combined a genome-wide DNA methylation analysis with inferential and predictive statistical approaches. We identified 149 robust differentially methylated regions between high-ranking and low-ranking females (rankDMRs). RankDMRs were found in genes associated with immune function, glutamate receptor signalling and energy conversion.
Research sample	We collected gut epithelium cells from faecal samples of 42 free-ranging female spotted hyenas (24 cubs and 18 adults) of either low (15 cubs, 9 adults) or high (9 cubs, 9 adults) social status.
Sampling strategy	Study animals were habituated to the presence of observers in vehicles. During each field session we routinely scored the presence of all clan members present within a radius of 100m of the communal den(s). We individually recognized clan members by their spot pattern and cubs by ear notches, scars or bald patches. We estimated cub age ± 7 days using pelage characteristics and locomotion. Cubs can be observed shortly after birth as they are nursed at the entrance to underground burrows. As in other previous studies we defined cubs as individuals younger than one year. We routinely collected faecal samples from clan members. Faecal samples were collected immediately after defecation and stored on cool packs in the field before freezing at our research station.<br>At our research station in the Serengeti NP, we collected mucus (containing gut epithelium cells) with a scalpel from faeces, and stored mucus samples with dimethyl sulfoxide (DMSO) or RNA Later at -10°C or in liquid nitrogen before shipment to Germany, where they were stored at -80°C until analysis. We carefully removed the whitish layer of mucus to minimise contamination with DNA from gut bacteria. For the current study we focused on females to avoid dealing with potential influences of sex on DNA methylation patterns and because we had more detailed information about the life histories of (philopatric) females than about males. We used 24 samples from cubs and 18 from adult females. Adult samples originated from nine low-ranking and nine high-ranking females (Supplementary Table S1). Cub samples originated from cubs of 15 low-ranking mothers and 9 high-ranking mothers. Cubs were aged between two and nine months (Supplementary Table S1).

Data collection	We determined the social rank of adult females using standard methods based on the observation of submissive acts in dyadic interactions recorded ad libitum and during focal observations. We recorded submissive behaviours during dyadic interactions among adult females and constructed strictly linear dominance hierarchies for each clan. Dominance hierarchies were adjusted after each loss or recruitment of adult females and when dyadic interaction data revealed that an individual had increased or fallen in rank. To compare ranks held by individuals within hierarchies containing different numbers of animals within and across clans, we calculated standardised ranks. This measure places the ranks within a given clan hierarchy evenly between the highest (standardised social rank: +1) and the lowest (standardised social rank: −1) rank. We classified adult females with standardised ranks above and below the median rank as high-ranking and low-ranking, respectively. Adult females have high probabilities of staying within a social status class (yearly probabilities of staying high-ranking and low-ranking were equal to 0.94 and 0.97, respectively. Even so, we verified that the females from which we collected samples had some variation in their standardised social ranks prior to sampling (Supplementary Figure 2a). We selected samples from females at the top and bottom of the hierarchy to maximise the chance of observing differences in DNA methylation patterns between high-ranking and low-ranking females. In our dataset, the 18 high-ranking females (9 cubs, 9 adults) had an average standardised social rank of 0.70 (ranging from +0.07 to +1) and the 24 low-ranking females (15 cubs, 9 adults) an average standardised social rank of -0.56 (ranging from -0.07 to -1) (Supplementary Figure 2A).<br><br>We assigned sampled cubs, i.e. individuals younger than one year, the social rank of their genetic mothers. During their long period of dependence on maternal milk, young spotted hyenas learn their social position, just below that held by their mothers in the clan hierarchy, by observing them interacting with other clan members and receiving social support from their mothers and closely related females. In another study population in Kenya, about 80% of cubs acquired the exact rank expected under such rules (maternal rank 'inheritance'). Previous research in our study population and others show that maternal social status influences milk transfer and offspring growth rate during the first six months, survival to adulthood, and the age at first reproduction. Cubs of high-ranking mothers were also less likely to be infected with canine distemper virus and once infected more likely to survive the infection. Thus, in both cubs and adults, social status influences life history traits and measures of reproductive performance.
Timing	We collected data and faecal samples on individually known spotted hyenas between 2009 and 2019 in the context of an ongoing, individual-based long-term research project on three clans located at the centre of the Serengeti NP (Isiaka clan: monitored since 1987, Pool: since 1989, Mamba: since 1990). During our routine field sessions, we observed hyenas in their clan territories at communal den sites during periods of several hours around dawn and dusk.
Data exclusions	n/a
Non-participation	n/a
Randomization	For unbiased treatment, we blinded the animals ranks until final data analysis.

Reporting for specific materials, systems and methods

We require information from authors about some types of materials, experimental systems and methods used in many studies. Here, indicate whether each material, system or method listed is relevant to your study. If you are not sure if a list item applies to your research, read the appropriate section before selecting a response.

Materials & experimental systems

n/a	Involved in the study	
☒	☐	Antibodies
☒	☐	Eukaryotic cell lines
☒	☐	Palaeontology and archaeology
☐	☒	Animals and other organisms
☒	☐	Clinical data
☒	☐	Dual use research of concern
☒	☐	Plants

Methods

n/a	Involved in the study	
☒	☐	ChIP-seq
☒	☐	Flow cytometry
☒	☐	MRI-based neuroimaging

Animals and other research organisms

Policy information about studies involving animals; ARRIVE guidelines recommended for reporting animal research, and Sex and Gender in Research

Laboratory animals	n/a
Wild animals	We collected gut epithelium cells from faecal samples of 42 free-ranging female spotted hyenas (24 cubs and 18 adults) of either low (15 cubs, 9 adults) or high (9 cubs, 9 adults) social status. All sample material was taken non-invasively.
Reporting on sex	Our results apply to spotted hyenas only with sex female.
Field-collected samples	We collected gut epithelium cells from faecal samples of 42 free-ranging female spotted hyenas (24 cubs and 18 adults) of either low (15 cubs, 9 adults) or high (9 cubs, 9 adults) social status. All sample material was taken non-invasively.
Ethics oversight	We conducted this study under research permits from the Tanzania Commission for Science and Technology and permission from

Ethics oversight	the Tanzanian National Parks Authority and Tanzanian Wildlife Research Institute. All procedures were performed in accordance with the Leibniz Institute for Zoo and Wildlife Research Ethics Committee on Animal Welfare (permit number: 2017-11-02).

Note that full information on the approval of the study protocol must also be provided in the manuscript.
